# Effect of Formic Acid on the Outdiffusion of Ti Interstitials at TiO_2_ Surfaces: A DFT+U Investigation

**DOI:** 10.3390/molecules27196538

**Published:** 2022-10-03

**Authors:** Daniel Forrer, Andrea Vittadini

**Affiliations:** 1 Istituto di Chimica della Materia Condensata e di Tecnologie per l’Energia del CNR (CNR-ICMATE), Via Marzolo 1, I-35131 Padova, Italy; 2 Dipartimento di Scienze Chimiche (DiSC), Università di Padova, Via Marzolo 1, I-35131 Padova, Italy

**Keywords:** titanium dioxide, interstitial defects, formic acid, density functional theory

## Abstract

Ti interstitials play a key role in the surface chemistry of TiO_2_. However, because of their elusive behavior, proof of their participation in catalytic processes is difficult to obtain. Here, we used DFT+U calculations to investigate the interaction between formic acid (FA) and excess Ti atoms on the rutile-TiO_2_(110) and anatase-TiO_2_(101) surfaces. The excess Ti atoms favor FA dissociation, while decreasing the relative stability of the bidentate bridging coordination over the monodentate one. FA species interact significantly with the Ti interstitials, favoring their outdiffusion. Eventually, Ti atoms can emerge at the surface forming chelate species, which are more stable than monodentate FA species in the case of rutile, and are even energetically favored in the case of anatase. The presence of Ti adatoms that can directly participate to surface processes should then be considered when formic acid and possibly carboxylate-bearing species are adsorbed onto TiO_2_ particles.

## 1. Introduction

Since the infancy of surface science, defects have attracted great attention because of their ability to modify the physical and chemical properties of solid surfaces [1]. In this regard, understanding the interactions of defects with adsorbed molecular species is particularly interesting, as it is relevant in chemical sensing and catalysis applications. Although these considerations are rather obvious, atomic level investigations of defect-based complexes became feasible only since the last decade of the past century, thanks to the improvements in the computational tools and experimental techniques such as scanning tunneling microscopy (STM), and photoelectron and vibrational spectroscopies. Early work was devoted to metals and semiconductors, also because these are ideally suited for STM, which allows for directly observing the surface species. More recently, these studies have been extended to semiconductors, and especially to metal oxides, which are important because they are suitable for a number of applications ranging from the fabrication of functional materials and devices to catalysis [2,3,4].

An important feature of STM is its sensitivity to the local density of states at the surface. This makes defects present at the first atomic layer, such as steps, adatoms, and vacancies, easily detectable, thus making an effective combination of experimental and computational investigations possible. However, some defects prefer to stay deeper in the bulk [5,6,7], i.e., practically invisible to STM, and yet they influence surface chemistry. This is the case for Ti interstitials at the TiO_2_ rutile and anatase surfaces, whose catalytic role in important processes, such as O_2_ activation, has been predicted by density functional theory (DFT) calculations [8].

It has been pointed out that O vacancies at the TiO_2_ surface are able to repel Ti interstitials and vice versa. Thus, because of the dearth of interstitials, vacancies turn out to dominate the redox chemistry of TiO_2_(110) [9]. However, this scenario may be altered by the presence of adsorbates. Au nanoparticles can induce the outward diffusion and surface segregation of interstitial Ti defects, even under the reducing condition with O vacancy [10]. Analogously, porphyrins are able to change the energy profile for the outdiffusion of Ti interstitials at r-TiO_2_(110) [11], so that these defects can emerge at the surface and can finally be captured by the adsorbed free bases (“self-metalation reaction”) even in mild conditions, as confirmed by STM and XPS [12,13]. A detailed analysis of the mininum energy paths reveals that the process is triggered by a nucleophilic attack of an iminic N atom to a six-fold coordinated Ti surface ion assisted by the outdiffusing Ti interstitial.

The above-given survey suggests that further investigations are needed to understand which species can attract Ti interstitials at the TiO_2_ surface, and how this can influence the TiO_2_ surface chemistry. In particular, the above-described mechanism of the porphyrin self-metalation reaction poses the question of whether other species may be able to extract and sequestrate Ti interstitials at TiO_2_ surfaces. Clearly, a stable coordination of Ti adatoms is most likely achieved by bi- or multi-dentate ligands. This prompted us to investigate the interaction of formic acid (FA), a prototypical bidentate ligand, with the most representative surfaces of the main TiO_2_ polymorphs, i.e., rutile (110) and anatase (101)—hereafter r-TiO_2_(110) and a-TiO_2_(101)—in the presence of excess Ti atoms.

For decades, the interaction of FA with ideal and defected TiO_2_ surfaces has been intensely investigated, as, on the one hand, FA is a probe to identify surface basic sites, and, on the other hand, carboxylic groups can anchor functional molecules at metal oxide surfaces. In general, FA can be adsorbed either in molecular or dissociated forms, and can be coordinated either to a single cation, in a bidentate chelating (BC) or in a monodentate (M) fashion, or to two cations in a bridging bidentate (BB) fashion. The species predicted to exist at the low-index TiO_2_ surfaces are the monodentate one, both in the molecular (MM) and in dissociated (MD) forms, and the BB one. The high stability of the BB form at the rutile (110) surface has been apparent since the earliest investigations [14]. This is confirmed by a number of experimental techniques, first of all the 2 × 1 pattern observed in LEED, which corresponds to the formation of arrays of formate ions coordinated to couples of adjacent five-fold coordinated (Ti-5c) cations [15,16]. A less clear picture emerges for FA adsorption at the anatase (101) surface. Early generalized gradient corrected (GGA) calculations have predicted the MM form to be slightly favored over the BB one [17], whereas recent work using SCAN meta-GGA or dispersion-corrected functionals found the opposite trend, with the BB form slightly favored over the MM one. Anyway, at the anatase (101) surface the BB configuration is not strongly favored over the monodentate one as at the rutile (110) surface [18,19,20]. This difference has been attributed to the larger distance between the Ti(5c) ions at the two surfaces [17].

Interestingly, FA is an effective vacancy healer at r-TiO_2_(110) [21]. This, on the one hand, justifies a model study where only formic acid and interstitials are considered, and, on the other hand, makes the study of the interaction between Ti interstitials and formic acid particularly attractive. Actually, Tanner et al. [8] showed, through HSE06 calculations, that for r-TiO_2_(110), FA molecules attract excess Ti atoms, substantially reducing the energy difference between the first layer and second layer interstitial sites. The formation of surface complexes where Ti interstitials are directly bonded to FA was not considered in that investigation. Concerning a-TiO_2_(101), vacancies stayed below the surface, and induced the dissociation of monodentate species, eventually favoring the BB coordination [18]. To the best of our knowledge, the interaction between Ti interstitials and formic acid at the a-TiO_2_(101) surface has not been investigated.

In this work, we used DFT+U calculations to understand whether formic acid is able to segregate Ti interstitials at the TiO_2_ surface. To this end, we considered the rutile (110) and the anatase (101) surfaces, which are the most abundant surfaces of the most stable polymorphs.

## 2. Results

### 2.1. Adsorption of Formic Acid at the Undefected Surfaces

**Figure 1:**
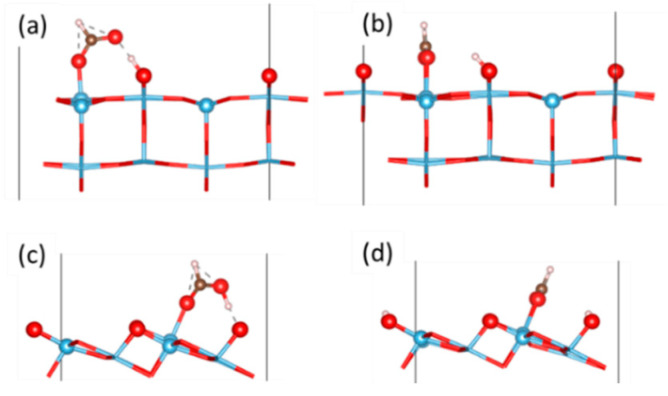
Side views for M and BB species at the rutile (110) surface (**a**,**b**) and at the anatase (101) surface (**c**,**d**). Blue: Ti; red: O; brown: C; white: H.

In agreement with recent investigations [18,20,22], our DFT+U (U = 3.5 eV) calculations predict that BB species are the most stable both at the anatase (101) and at the rutile (110) surfaces, while the adsorption energy is considerably stronger for rutile than for anatase (−1.79 eV vs. −1.38 eV). Concerning the monodentate form, the MD species (Δ*E*_ads_ = −1.30 eV) is preferred for rutile, whereas the MM one is favored for anatase (−1.20 eV). The easier dissociation at the rutile (110) surface is explained by the higher basicity of the O(2c) ions. On the other hand, the particular stability of the BB at the rutile surface can be ascribed to a concurrent structural effect, as the Ti(5c)-Ti(5c) distance at rutile (110) is particularly suited to the “byte” of the formate ion [17]. Side views of the species are shown in Figure 1.

### 2.2. Sites for Excess Ti Atoms at the Rutile Surface

**Figure 2:**
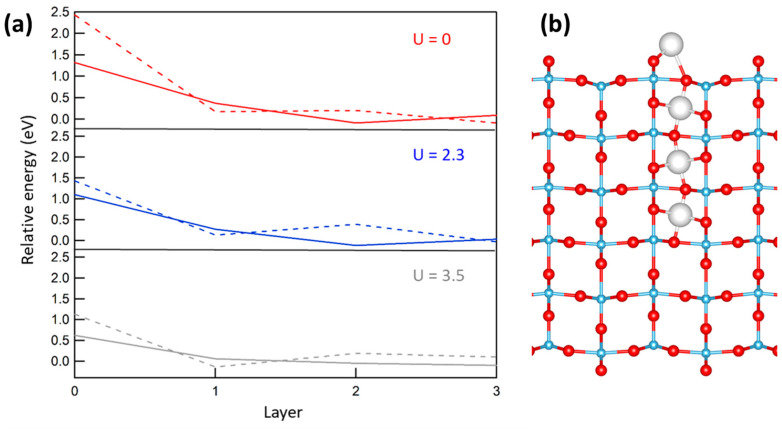
(**a**) Relative energy for an excess Ti atom at the TiO_2_(110) surface as a function of the occupied layer. Layer = 0 stands for adatom sites. Solid lines indicate A-type sites, dashed lines indicate B-type sites (see text). (**b**): lateral view of the slab, where A-type interstitial sites are shown as large grey spheres. U values in eV.

We first examined the stability of various sites on the clean surfaces of r-TiO_2_(110). We considered two adatom sites, A and B [7], where the excess Ti was three-fold coordinated to two O(2c) atoms and to one/two O(3c) atoms, respectively. Furthermore, we considered the sub-surface octahedral interstitial sites approximately placed below the A (see Figure 2b) and B adatom sites, hereafter labeled as L*n*-A and L*n*-B, respectively, where *n* = 1, 2, and 3.

The relative energies of all of the investigated sites is reported in Figure 2. We first note that the energy decreased by moving the Ti excess atom deeper into the subsurface. At the surface, the A site was always more stable than the B one, whereas A- and B-type sites were alternately favored when considering the increasingly deeper layers. In particular, results with U = 3.5 eV indicate that the A adatom was unfavored by 0.56 eV with respect to the first-layer L1-A interstitial, which was in turn unfavored by 0.11 eV compared with the second-layer L2-A site. These results are in good agreement with those reported in [7]. We also note that the difference between A- and B-type sites as very small at the third layer, according to the equivalence of the sites in the bulk. Finally, we remark that, upon increasing the value of the U parameter, the adatom sites were considerably stabilized, i.e., the outdiffusion of the interstitials was predicted to be easier. In fact, the energy required to move a Ti atom from the L3-A interstitial site to the A adatom site passed from 1.3 eV (U = 0) to 1.0 V (U = 2.3 eV) and finally to just 0.5 eV in the U = 3.5 eV case.

### 2.3. Adsorption of Formic Acid at the Ti-Rich TiO_2_(110) Surface

**Figure 3:**
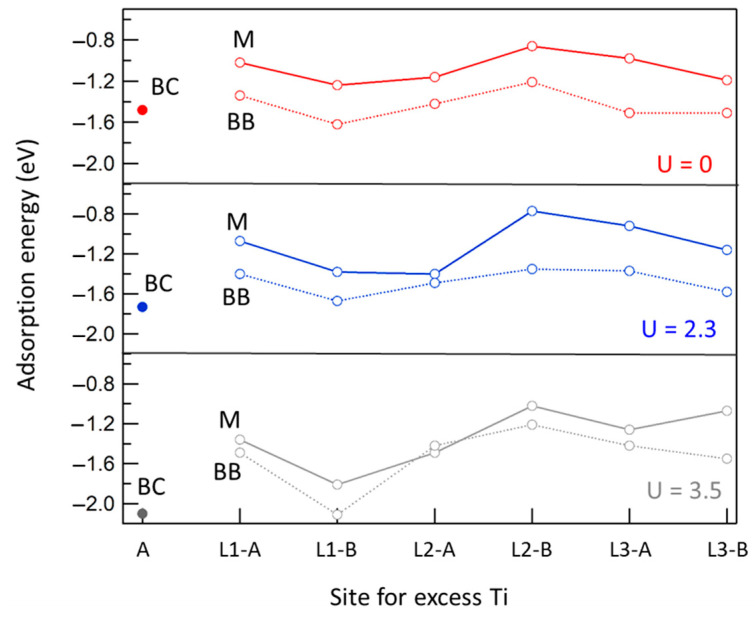
Adsorption energy at the TiO_2_(110) surface for formic acid in the monodentate form (M, solid line) and in the bridging bidentate form (BB, dotted line) as a function of the site for an excess TI atom (see text). BC indicates a formate chelating species. U values in eV.

Here, we consider the interaction between FA and excess Ti atoms. As we can combine various FA adsorption modes with each of the above described sites for the excess Ti atom, we need to compare the stability of several complexes. To this end, we report in Figure 3 the energy differences Δ*E*, computed as follows:

Δ*E*[FA@X] = E[FA@X] − E[X*] − E[FA(g)] (1)

where FA@X indicates a slab with an FA adsorbed at the surface where the excess Ti atom is located at a given X site; X* indicates a slab representing a clean surface with the excess Ti atom located at the most stable site, i.e., for each value of U the absolute minimum in the pertaining panel of Figure 2. FA(g) is a molecule in the gas phase, placed in a large cubic supercell. Subsurface sites have been considered only for M and BB species, while only the A adatom site has been considered for the BC species. In any case, the Ti interstitial site has been chosen to be as close as possible to the FA molecule. Such energy differences can be interpreted as the sum of the adsorption energy of FA plus the energy required to move the Ti excess atom from its most stable position to the named site, but can also be read simply as relative energies, where the reference state is the FA in the gas phase and the slab with the Ti excess atom at the most stable site.

**Figure 4:**
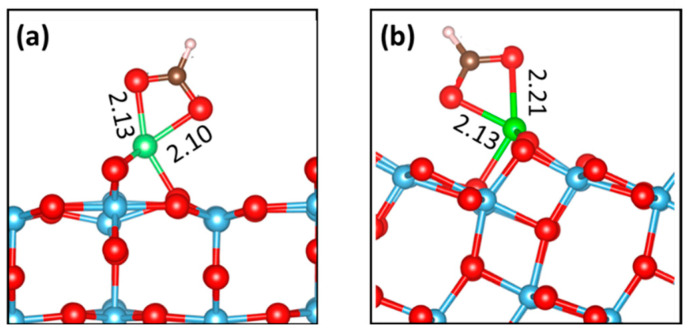
Side sketches of Ti adatoms coordinated by a bidentate chelating formate anion at (**a**) the rutile surface and (**b**) at the anatase surface. Atom colors are the same as in Figure 1. The Ti adatom is shown as a green sphere. Distances in Å refer to calculations with U = 3.5 eV.

The results show the following: (*i*) When the excess Ti atom is in an interstitial position, the BB form is generally favored, but the energy difference with regards to the MD species (U = 3.5 eV) tends to be smaller than in the undefected surface case. (*ii*) The adsorption energies increase with U. (*iii*) The alternate stability of the A and B sites while going deeper in the slab is retained. (*iv*) The presence of adsorbed FA species facilitates the diffusion of Ti interstitials from the bulk to the first sub-surface layer (L1-B site), in line with the HSE06 calculations by Tanner et al. [8]. (*v*) Quantitatively, this effect also depends on the choice of the U parameter: in the case of U = 3.5 eV, the BB species interacting with a Ti at a L1-B site has a −2.20 eV adsorption energy, which is at least ~0.5 eV more stable than a BB species interacting with a third-layer interstitial. (*vi*) The adatom site is strongly stabilized by the BC species, whatever the U value. Actually, the BC-Ti@A complex is in any case more stable than the M-Ti@L1B one, and is almost as stable as BB-Ti@L1B. A sketch of the BC-adatom complex is reported in Figure 4a.

### 2.4. Sites for Excess Ti Atoms at the Anatase Surface

**Figure 5:**
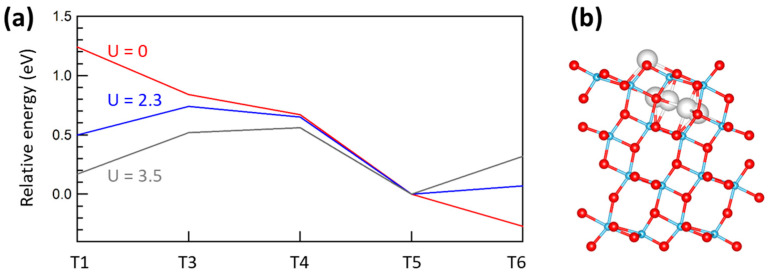
(**a**) Relative energy for an excess Ti atom at the a-TiO_2_(101) surface as a function of the occupied site, Tn. (**b**) Lateral view of the slab showing the positions of the examined interstitial sites as grey spheres. Colors are the same as in Figure 4. U values in eV.

We considered an adatom site, and four increasingly deep sub-surface sites, as depicted in Figure 5, right. The sites were labeled as T1, T3, T4, T5, and T6, following a previously introduced notation [23]. The T6 site was energetically close to a bulk site [22]. The T1 adatom bridged two neighboring O(2c) anions, and was also coordinated to two O(3c) ions. We disregarded a further adatom site (T2), as it was highly unfavored energetically [23]. As for rutile, energies were referred to the most stable site, which was T5, for the DFT+U calculations. We also computed defect formation energies, whose GGA values were within 0.1 eV, which agreed with those of [23]. For instance, for the T1 adatom, we computed *E* = 8.94 eV, to be compared with the 8.87 eV of the literature. Overall, our results indicate that deeper interstitial sites were favored with respect to the adatom site, but this order was partially inverted by increasing U (see Figure 5). In comparison with rutile, this effect was similar but stronger, so that the T1 adatom was more stable than T3 and T4 for U = 2.3 eV, and was almost as stable as T5 for U = 3.5 eV.

### 2.5. Adsorption of Formic Acid at the Ti-Rich a-TiO_2_(101) Surface

**Figure 6:**
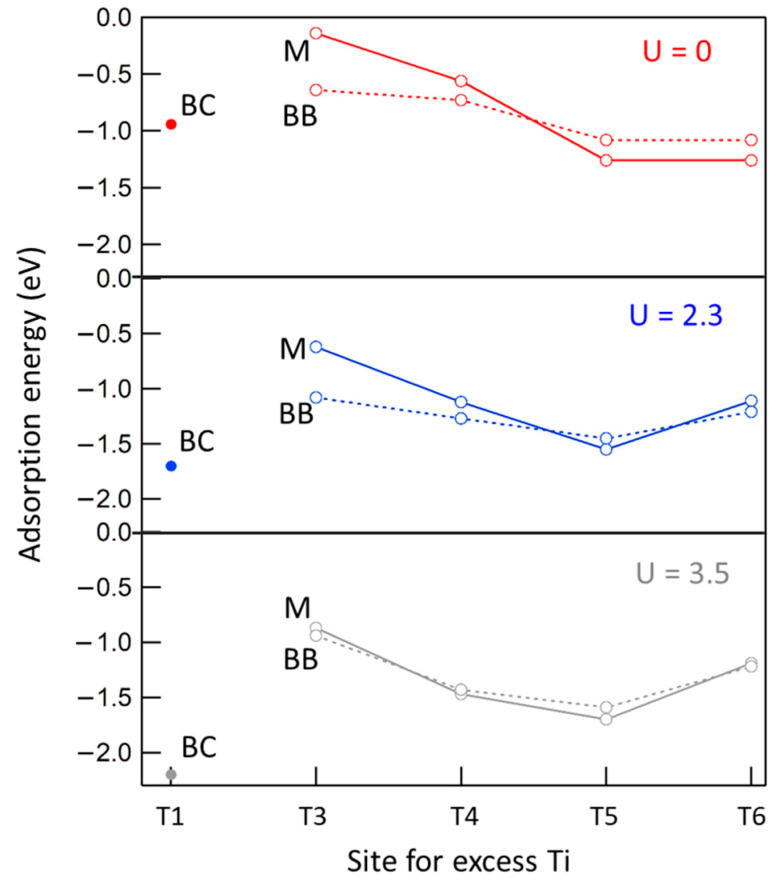
Adsorption energy at the a-TiO_2_(101) surface for formic acid in the monodentate form (M, solid line) and in the bidentate form (BB, dotted line) as a function of the site for the excess Ti atom. BC indicates a bidentate chelating species where the excess Ti is an adatom.

Here, we examine FA adsorption at the Ti-doped anatase (101) surface. Note that the presence of Ti interstitials induced the dissociation of monodentate FA. Interestingly, a similar effect has also been predicted when O vacancies are present at the (101) surface [18]: this is not surprising, as both Ti interstitials and O vacancies give rise to surface reduction. As in the rutile case, we evaluated the stabilities of a number of complexes, which are displayed in Figure 6. Once more, the results were influenced by the choice of the U parameter. In fact, increasing U not only strengthened the adsorption energy, as for r-TiO_2_(110), but it also changed the preferred coordination of the formate species. In particular, GGA calculations always predict the M form (either molecular or dissociated, in the case of the reduced surface) to be most stable. For DFT+U calculations, the dissociated BB form was favored for the undefected system, whereas the M and the BB forms were similarly stable in the presence of Ti interstitials. Furthermore, the FA capability of attracting interstitials from the bulk to the subsurface sites seemed to be weaker when compared with the rutile case: the T5 site was actually preferred for both the clean surface and in the presence of adsorbed M and BB species. Interestingly, however, the BC-Ti@T1 complex (see Figure 4, right) was predicted to be more stable by at least 0.6 eV, compared with any other complexes, when using the DFT+U (U = 3.5 eV) calculations. In other words, FA could extract Ti interstitials from the anatase surface, sequestrating them under the form of the chelate complexes.

## 3. Discussion

The above-described results indicate that adsorbed FA species are able to attract Ti interstitials at the r-TiO_2_(110) and a-TiO_2_(101) surfaces, eventually forming chelate complexes where the interstitials are converted into adatoms. In the rutile case, the chelate form is more stable than the monodentate species, whatever site is considered for the Ti interstitial and for any U value. For anatase, the chelate form is the most stable species, at least for high U values. The particular stability of the BC form at the anatase surface is, at least in part, due to the pseudo-octahedral coordination assumed by the Ti adatom, where a three-fold coordinated O(3c) ion occupies the position opposite the lower chelating O atom. In fact, this O atom is significantly displaced towards the adatom, reaching a distance of 2.46 Å. In the rutile case, a less favorable five-fold coordination is instead obtained.

The formation of chelates at low-index TiO_2_ surfaces is an unprecedented finding, and is, in our opinion, quite relevant, as these complexes may play a role as catalytic centers, e.g., in oxygen activation or formic acid decomposition. They could be also involved in the functionalization of TiO_2_ nanoparticles. We could wonder why, contrary our predictions, chelate complexes have not been so far detected at r-TiO_2_(110) or at a-TiO_2_(101). Concerning rutile, two species have been detected by infrared reflection absorption spectroscopy (IRRAS) [22]. The majority of species clearly correspond to BB formates, whereas the minority species, whose molecular plane is rotated by 90° with regards to BB formates, are more difficult to identify. Both MD species and bidentate species simultaneously interacting with a Ti(5c) center and with an O vacancy have been proposed as models for the minority species in order to explain the observed orientation. The former model was finally chosen because the formation of minority species was not influenced by the vacancy concentration. However, the proposed BC model would not be ruled out by these criteria, and provides an alternative mode for minority FA species. The case of anatase, for which our results indicate that the BC complex should be the majority species, should, however, be examined more carefully. In this regard, we point out that the interstitial outdiffusion and capture is an activated process, which requires careful investigation of the energetics. In the case of tetraphenylporphyrin (2H-TPP) adsorbed at TiO_2_(110), where strong theoretical and experimental evidence for interstitial Ti extraction has been obtained, the onset for Ti sequestration is ~100 °C, while the computed barrier for the outdiffusion/metalation process is 0.65 eV. In the present case, we expect higher barriers, and thus a higher onset temperature. In fact, as already pointed out, M and BB species are not able to attract significantly sub-surface Ti interstitials, and the driving force for the capture process is lower than in the 2H-TPP case. In addition, Ti capture requires a change in FA coordination (from BB to BC), which is not required in the 2H-TPP/TiO_2_ (110) self-metalation process. Actually, in the most recent and accurate study, where DFT calculations have been combined with scanning tunneling microscopy, IRRAS, and electron-stimulated desorption experiments, only MD and BB species have been observed. However, these experiments have been carried out at temperatures (80–240 K) that are certainly too low to allow for the extraction of Ti interstitials. Xu et al. [24] performed IRRAS experiments on over annealed surfaces at temperatures ranging from 300 K to 500 K, and found no IR bands typical of BC species, but they did not rule out their presence in small amounts.

## 4. Computational Methods and Models

We used a computational approach based on density functional theory (DFT), which has been successfully adopted to study the outdiffusion of Ti interstitials at TiO_2_ surfaces both in the absence [7] and in the presence of adsorbates [10,11]. To this end, we adopted the PWSCF code of the QUANTUM ESPRESSO suite (QE) [25,26]. Valence orbitals were expanded on a plane–wave basis set with a kinetic energy cutoff of 25 Ry, while the cutoff on the augmentation density was 200 Ry. The PBE [27] exchange–correlation functional was adopted, including dispersion interactions by means of the D2 Grimme method [28,29]. The interaction between ion cores and valence electrons was modelled using ultrasoft pseudopotentials [30], whose core include 1s orbitals for C, N, and O, and 1s-2p orbitals for Ti. Hubbard U corrections are used to treat Ti d states [31,32,33]. In past investigations of Ti interstitials in TiO_2_, various U values have been used, such as 2.5 eV [34], 3 eV [35], and 4.2 eV [36]. In this regard, we found that high U values reproduce the energy of the gap states energy better, but U values in the 2–3 eV range were more appropriate to describe the redox processes involving TiO_2_ [37]. For instance, U = 2.3 eV reproduces the experimental reduction energy of TiO_2_ to Ti_2_O_3_ quite well. On this basis, we used three U parameters, including U = 0.0 eV, which corresponds to plain GGA, as well as U = 2.3 eV and U = 3.5 eV.

Surfaces were modelled by means of a repeated slab approach, adopting the PBE-D2 theoretical lattice constants [38]. For the rutile (110) surface, we used a 4 × 2 supercell including six TiO_2_ layers (288 atoms), while for anatase (101), we used a 3 × 1 supercell including four TiO_2_ layers (144 atoms). The surface size of the two slabs were similar, both including four O(2c) and four Ti(5c) ions, whereas the thickness of the rutile slab was higher, because of well-known convergence problems [39]. Adsorbates were placed only on the top surface of the slab. All of the atoms were optimized for clean surfaces, whereas the two bottom TiO_2_ layers were then kept fixed throughout the subsequent calculations.

## 5. Conclusions

We carried out DFT+U calculations to study the interaction between formic acid and excess Ti atoms at the most stable surfaces of the anatase and rutile TiO_2_ polymorphs. In both cases, the presence of interstitials strengthened the relative stability of the monodentate species with respect to the bridging bidentate ones. It also caused a general increase in adsorption energies. An interesting aspect is the capability of the adsorbate to attract interstitials at the surface, which, in the case of monodentate and bidentate bridging species, is rather weak, especially for anatase. In contrast with this, bidentate chelating species can segregate interstitials at the surface quite effectively. In fact, calculations with U = 3.5 eV predicted that the chelate complex with the adatom complex was more stable than monodentate complexes with interstitials for the rutile, while it was 0.6 eV more stable than any other complex for anatase.

Overall, our results show that in the presence of formic acid, Ti interstitials can emerge at the TiO_2_ surface. These can in turn directly participate in surface chemical processes, as recently shown for other species, such as gold clusters and tetrapyrrole macrocycles. It is likely that similar phenomena can occur also for adsorbates using carboxylate as anchoring groups, which are commonly used to functionalize metal oxide nanoparticles.

## References

[1] Wandelt K. (1991). Properties and Influence of Surface Defects. Surf. Sci..

[2] Chen X., Mao S.S. (2007). Titanium Dioxide Nanomaterials:  Synthesis, Properties, Modifications, and Applications. Chem. Rev..

[3] Danish M.S.S., Bhattacharya A., Stepanova D., Mikhaylov A., Grilli M.L., Khosravy M., Senjyu T. (2020). A Systematic Review of Metal Oxide Applications for Energy and Environmental Sustainability. Metals.

[4] Kazachenko A., Vasilieva N., Fetisova O., Sychev V., Elsuf’ev E., Malyar Y., Issaoui N., Miroshnikova A., Borovkova V., Kazachenko A. (2022). New Reactions of Betulin with Sulfamic Acid and Ammonium Sulfamate in the Presence of Solid Catalysts. Biomass Convers. Biorefinery.

[5] Bennett R.A., McCavish N.D. (2005). Non-Stoichiometric Oxide Surfaces and Ultra-Thin Films: Characterisation of TiO_2_. Top. Catal..

[6] Finazzi E., Di Valentin C., Pacchioni G. (2009). Nature of Ti Interstitials in Reduced Bulk Anatase and Rutile TiO_2_. J. Phys. Chem. C.

[7] Mulheran P.A., Nolan M., Browne C.S., Basham M., Sanvillee E., Bennett R.A. (2010). Surface and Interstitial Ti Diffusion at the Rutile TiO_2_(110) Surface. Phys. Chem. Chem. Phys..

[8] Tanner A.J., Wen B., Ontaneda J., Zhang Y., Grau-Crespo R., Fielding H.H., Selloni A., Thornton G. (2021). Polaron-Adsorbate Coupling at the TiO_2_(110)-Carboxylate Interface. J. Phys. Chem. Lett..

[9] Yoon Y., Du Y., Garcia J.C., Zhu Z., Wang Z.-T., Petrik N.G., Kimmel G.A., Dohnalek Z., Henderson M.A., Rousseau R. (2015). Anticorrelation between Surface and Subsurface Point Defects and the Impact on the Redox Chemistry of TiO_2_(110). ChemPhysChem.

[10] Xia G.-J., Lee M.-S., Glezakou V.-A., Rousseau R., Wang Y.-G. (2022). Diffusion and Surface Segregation of Interstitial Ti Defects Induced by Electronic Metal–Support Interactions on a Au/TiO_2_ Nanocatalyst. ACS Catal..

[11] Kremer M.K., Forrer D., Rogero C., Floreano L., Vittadini A. (2021). Digging Ti Interstitials at the R-TiO_2_(110) Surface: Mechanism of Porphyrin Ti Sequestration by Iminic N Nucleophilic Attack. Appl. Surf. Sci..

[12] Koebl J., Wang T., Wang C., Drost M., Tu F., Xu Q., Ju H., Wechsler D., Franke M., Pan H. (2016). Hungry Porphyrins: Protonation and Self-Metalation of Tetraphenylporphyrin on TiO_2_(110)-1 × 1. ChemistrySelect.

[13] Lovat G., Forrer D., Abadia M., Dominguez M., Casarin M., Rogero C., Vittadini A., Floreano L. (2017). On-Surface Synthesis of a Pure and Long-Range-Ordered Titanium(IV)-Porphyrin Contact Layer on Titanium Dioxide. J. Phys. Chem. C.

[14] Bates S.P., Kresse G., Gillan M.J. (1998). The Adsorption and Dissociation of ROH Molecules on TiO_2_(110). Surf. Sci..

[15] Chambers S.A., Henderson M.A., Kim Y.J., Thevuthasan S. (1998). Chemisorption Geometry, Vibrational Spectra, and Thermal Desorption of Formic Acid on TiO_2_(110). Surf. Rev. Lett..

[16] Hayden B.E., King A., Newton M.A. (1999). Fourier Transform Reflection−Absorption IR Spectroscopy Study of Formate Adsorption on TiO_2_(110). J. Phys. Chem. B.

[17] Vittadini A., Selloni A., Rotzinger F.P., Grätzel M. (2000). Formic Acid Adsorption on Dry and Hydrated TiO_2_ Anatase (101) Surfaces by DFT Calculations. J. Phys. Chem. B.

[18] Wang Y., Wen B., Dahal A., Kimmel G.A., Rousseau R., Selloni A., Petrik N.G., Dohnálek Z. (2020). Binding of Formic Acid on Anatase TiO_2_(101). J. Phys. Chem. C.

[19] Kwon S., Lin T.C., Iglesia E. (2020). Elementary Steps and Site Requirements in Formic Acid Dehydration Reactions on Anatase and Rutile TiO_2_ Surfaces. J. Catal..

[20] Tabacchi G., Fabbiani M., Mino L., Martra G., Fois E. (2019). The Case of Formic Acid on Anatase TiO_2_(101): Where Is the Acid Proton?. Angew. Chem. Int. Ed..

[21] Hu S., Bopp P.A., Österlund L., Broqvist P., Hermansson K. (2014). Formic Acid on TiO_2–x_ (110): Dissociation, Motion, and Vacancy Healing. J. Phys. Chem. C.

[22] Mattsson A., Hu S., Hermansson K., Österlund L. (2014). Adsorption of Formic Acid on Rutile TiO_2_ (110) Revisited: An Infrared Reflection-Absorption Spectroscopy and Density Functional Theory Study. J. Chem. Phys..

[23] Cheng H., Selloni A. (2009). Energetics and Diffusion of Intrinsic Surface and Subsurface Defects on Anatase TiO_2_(101). J. Chem. Phys..

[24] Xu M., Noei H., Buchholz M., Muhler M., Wöll C., Wang Y. (2012). Dissociation of Formic Acid on Anatase TiO_2_(101) Probed by Vibrational Spectroscopy. Catal. Today.

[25] Giannozzi P., Baroni S., Bonini N., Calandra M., Car R., Cavazzoni C., Ceresoli D., Chiarotti G.L., Cococcioni M., Dabo I. (2009). QUANTUM ESPRESSO: A Modular and Open-Source Software Project for Quantum Simulations of Materials. J. Phys. Condens. Matter.

[26] Giannozzi P., Andreussi O., Brumme T., Bunau O., Nardelli M.B., Calandra M., Car R., Cavazzoni C., Ceresoli D., Cococcioni M. (2017). Advanced Capabilities for Materials Modelling with QUANTUM ESPRESSO. J. Phys. Condens. Matter.

[27] Perdew J., Burke K., Ernzerhof M. (1996). Generalized Gradient Approximation Made Simple. Phys. Rev. Lett..

[28] Grimme S. (2006). Semiempirical GGA-Type Density Functional Constructed with a Long-Range Dispersion Correction. J. Comput. Chem..

[29] Barone V., Casarin M., Forrer D., Pavone M., Sambi M., Vittadini A. (2009). Role and Effective Treatment of Dispersive Forces in Materials: Polyethylene and Graphite Crystals as Test Cases. J. Comput. Chem..

[30] Vanderbilt D. (1990). Soft Self-Consistent Pseudopotentials in a Generalized Eigenvalue Formalism. Phys. Rev. B.

[31] Anisimov V.I., Aryasetiawan F., Lichtenstein A.I. (1997). First-Principles Calculations of the Electronic Structure and Spectra of Strongly Correlated Systems: The LDA + U Method. J. Phys. Condens. Matter.

[32] Anisimov V.I., Zaanen J., Andersen O.K. (1991). Band Theory and Mott Insulators: Hubbard U Instead of Stoner I. Phys. Rev. B.

[33] Himmetoglu B., Floris A., de Gironcoli S., Cococcioni M. (2014). Hubbard-Corrected DFT Energy Functionals: The LDA+U Description of Correlated Systems. Int. J. Quantum Chem..

[34] Stausholm-Møller J., Kristoffersen H., Hinnemann B., Madsen G., Hammer B. (2010). DFT+U Study of Defects in Bulk Rutile TiO_2_. J. Chem. Phys..

[35] Nolan M., Elliott S.D., Mulley J.S., Bennett R.A., Basham M., Mulheran P. (2008). Electronic Structure of Point Defects in Controlled Self-Doping of the TiO_2_ Surface: Combined Photoemission Spectroscopy and Density Functional Theory Study. Phys. Rev. B.

[36] Morita K., Shibuya T., Yasuoka K. (2017). Stability of Excess Electrons Introduced by Ti Interstitial in Rutile TiO_2_(110) Surface. J. Phys. Chem. C.

[37] Lutfalla S., Shapovalov V., Bell A.T. (2011). Calibration of the DFT/GGA+U Method for Determination of Reduction Energies for Transition and Rare Earth Metal Oxides of Ti, V, Mo, and Ce. J. Chem. Theory Comput..

[38] Forrer D., Vittadini A. (2011). 2D vs. 3D Titanium Dioxide: Role of Dispersion Interactions. Chem. Phys. Lett..

[39] Bredow T., Giordano L., Cinquini F., Pacchioni G. (2004). Electronic Properties of Rutile TiO_2_ Ultrathin Films: Odd-Even Oscillations with the Number of Layers. Phys. Rev. B.

